# Comparative evaluation of ferrule designs regarding fracture resistance about crowned anterior teeth using prefabricated post and composite core under direct loading: An in vitro study

**DOI:** 10.6026/973206300213582

**Published:** 2025-10-31

**Authors:** Smritashree Baruah, Polysmita Ojah, Sangita Nath, Shailesh Jain, Nikhil P. Agnihotri, Surabhi Duggal

**Affiliations:** 1 Department of Prosthodontics and Crown and Bridge, Tezpur Medical College and Hospital, Tezpur, Assam, India; 2 Department of Prosthodontics and Crown and Bridge, Government Dental College, Srimanta Sankaradeva University of Health Sciences, Dibrugarh, Assam, India; 3 Department of Prosthodontics and Crown and Bridge, Bhojia Dental College, Baddi, Himachal Pradesh, India; 4 Department of Prosthodontics and Crown and Bridge, DJ College of Dental Sciences and Research, Modinagar, Uttar Pradesh, India; 5 Department of Prosthodontics and Crown and Bridge, School of Dental Sciences, Sharda University, Greater Noida, Uttar Pradesh, India

**Keywords:** Fracture resistance, ferrule, sodium hypochlorite, prefabricated glass fiber, composite core

## Abstract

The effect of different ferrule designs on the fracture resistance of endodontically treated maxillary central incisors. Caries-free maxillary central incisors were endodontically treated and divided into five groups (n=12) based on ferrule design. All teeth were restored with prefabricated glass fiber posts, composite cores and metal crowns, then subjected to compressive loading using a universal testing machine. The group with a 2 mm circumferential ferrule showed the highest fracture resistance, while the group with no ferrule exhibited the lowest. Statistically significant differences were found among most groups, except between Group 2 and Group 3 (p=0.025).

## Background:

Endodontically restored anterior teeth with ample damage to tooth structure frequently demands a post and core due to sideward and shearing force acting on it and presence of compact pulp chambers as compared to molars [1]. The post is a comparatively stiff material extending about two-thirds of the length of the root channel of the pulpless teeth that provides augmentation and retention. The core is the coronal adjunct of the post, which restores the lost coronal tooth structure. The retention for the final restoration is furnished by the core and the core is retained by the dowel [2]. When a post and core is advisable, ferrule is the crucial constituent in the tooth preparation [3]. The term "ferrule" is derived from the Latin word, "ferrum", meaning iron and "viriola", meaning a bracelet. It serves as an encircling band of cast metal around the coronal surface of the tooth providing protective reinforcement to root filled teeth. The notion of ferrule was initially put forth by Rosen in 1961. In literature, the ferrule was designated by various names such as extra coronal brace, metal band, metal collar and subgingival collar and subgingival apron of gold [4]. Sorensen and Engelman modified the definition of ferrule as 360 degree metal collar of the crown surrounding the parallel walls of the dentin extending coronal to the shoulder of the preparation [5]. The ferrule serves various purposes. It cuts back on the wedging effect of the tapered dowel combats the functional lever forces and the lateral forces exerted during dowel insertion and enhance resistance to vigorous occlusal burden [6]. Additionally, the cervical brim of the cemented crown that surrounds the remaining dental structure accounts for the so-called concept of "ferrule effect" [5, 7].

The ferrule effect distributes the forces on the remaining tooth structure, reduces the disruption of the cementing agent of the post-core or crown towards the tooth and decreases the possibility of root breakdown thereby enhancing the integrity of the tooth and increasing its mechanical strength [5, 8]. Contradictory results have been reported when the effect of post-and-core materials on the fracture strength of endodontically treated teeth has been investigated in vitro [8, 9, 10- 11]. Prefabricated posts have shown lower fracture strength than cast post-and-cores, but their failure mode may allow repair of the restoration [12, 13]. However, more recent studies have reported that the placement of fiber posts also leads to catastrophic fractures [12, 14]. In the restoration of an anterior endodontically treated tooth with at least 2mm or more of remaining coronal dentine, a post provides no statistically significant improvement in fracture resistance compared to a similar tooth being restored with a radicular extension of 4mm of the composite core and no post [15]. Comprehension of the biomechanical performance of one ferrule design over another is important. Also there is sparse data about the partial ferrule design when considering the relationship between number, location and height of the remaining walls and how they impact the fracture resistance [16, 17]. Therefore, it is of interest to show the outcome of complete uniform and non-uniform ferrule designs (2 mm, 2 mm on buccal and palatal with 0.5mm proximal ferrule, 2mm on proximal with 0.5mm on buccal and palatal ferrule, no ferrule, 4mm composite extending into canal) on fracture resistance of endodontically treated teeth restored with glass fiber posts, composite core and subjected under compressive loading.

## Materials and Methods:

The study consisted of 60 extracted, caries-free, intact human maxillary central incisors that were selected and was carried out in the Department of Prosthodontics and Crown and Bridge. Sample size was calculated by using F test- ANOVA. During the selection of teeth, measurements were made with caliper to measure root length of at least 13 mm and almost similar buccolingual dimensions. The selected teeth were immersed in 3% sodium hypochlorite for 15 mins to remove any debris and soft tissue and washed in running tap water. Any stains present were mechanically removed with an ultrasonic scaler. The samples were then stored in saline until use. Working length of teeth was established by introducing an ISO size 10 stainless steel K file into the canal until visible at the apex. Working length was determined by deducting 1mm from the length recorded when file was visible at the apex. The cleaning and shaping of the canal was done with protapersSX,S1,S2. Irrigation was performed using 3% NaOCl solution in a 3ml syringe with 26 gauge needle after every change in file size. After completion of instrumentation, canals were finally rinsed with 10 ml of 17% EDTA, followed by 10ml of distilled water to remove any residue of sodium hypochlorite. The root canals were dried properly with absorbent paper points prior to obturation. Guttapercha 6% cone was inserted into the canal till the predetermined working length and "tug back" was felt. Sealer was mixed and applied to the walls of the canals with lentulospirals. Apical part of the guttapercha cone was coated with the sealer and introduced into the canal till the working length. The gutta perch was removed use a heated plugger at the orifice leaving 2mm of access opening to seal the coronal part of the canal. The canals were restored with temporary cement and placed in saline. Radiographs were taken to confirm the patency of the root canal filling.

After completion of endodontic procedure, the teeth were divided randomly into 5 groups of 12 teeth each (Figure 1A - see PDF).

[1] Group 1 - 2mm circumferential ferrule

[2] Group 2 - 2mm ferrule buccally and lingually, 0.5mm ferrule proximally

[3] Group 3 - 0.5mm ferrule buccally and lingually, 2mm ferrule proximally

[4] Group 4 - 1mm wide contralateral bevel with composite extending 4mm into the canal

[5] Group 5 - no ferrule

## Post space preparation and post cementation:

The teeth of group 1, 2, 3 and 4 were decoronated 2mm above the cementoenamel junction and those of group 5 were decoronated at the level of the CEJ using a diamond disc. The post spaces were prepared using peeso reamers no 1, 2, 3 and 4 sequentially to a length of 8mm leaving behind 4-5 mm of guttapercha at the apical end of the canal. Prefabricated glass fiber posts No. 2 (Angelus reforpost) were selected and radiographs were taken to check the proper extension of the post in the post space and to determine whether there was more than 1mm of remaining root dentin around the post. The post channel was air dried. Resin luting cement was dispensed on a paper pad and mixed using agate spatula, coated on the posts and cemented into the space.

## Core builds up:

An etchant 37% phosphoric acid was applied on the remaining tooth structure for 15 secs, rinsed and dried. Dentin bonding agent was applied and cured for 20 secs. Composite was build up in increments to complete the core, each increment requiring a photo-polymerization of 40 secs (Figure 1B - see PDF).

## Tooth preparation:

The conventional technique of tooth preparation was carried out using a high-speed air rotor hand piece and a diamond bur. A chamfer finish line of 1mm was prepared with a torpedo diamond. The incisocervical length of the core was retained to 6mm.

## Mounting of teeth:

The teeth were then mounted on acrylic resin block with long axis of each tooth parallel to long axis of block and the mid facial extent of cementoenamel junction located 2 mm coronal to resin block.

## Preparation of crown:

The crown patterns were prepared using inlay wax and PKT instruments. The patterns were invested in phosphate bonded investment (Bego-Belasun, Germany) in a casting ring. Casting was done using Ni-Cr alloy in an induction casting machine. The casting was retrieved and sandblasted to remove the residue. The crowns were finished, polished and cemented using glass ionomer luting cement. Following cementation, a lingual notch was scraped 3 mm below the incisal edge corresponding to the middle of the mesio-distal width to create a standard loading point (Figure 1C - see PDF).

## Testing of specimens:

The fracture resistance of the mounted specimens was checked with a universal testing machine. The machine applied controlled loads to the crown, 3 mm from its incisal edge on the palatal side at an angle of 135° to the long axis of the root. The testing was set at a cross head speed of 2.5mm/min. Failure threshold was defined as the point at which a specimen could no longer withstand increasing load and failure of post and core complex or root occurred. The data were then tabulated and sent for statistical analysis (Figure 1D - see PDF). Mean fracture resistance in Group 1(2 mm circumferential ferrule) was 1068.67 ± 107.89 , in Group 2(2 mm Buccal and lingual 0.5 mm proximal) was 730.92 ± 82.65,in Group 3(0.5 mm Buccal and lingual 2 mm proximal) was 652.08 ± 71.2, in Group 4(1 mm contra bevel with 4 mm composite into canal) was 853.00 ± 69.76 and in Group 5 (No ferrule) was 402.25 ± 83.16.Mean fracture resistance in Group 1(2 mm circumferential ferrule) was maximum as compared to other groups and minimum in Group 5 (No ferrule) (Table 1 - see PDF). There was highly significant difference in mean fracture resistance in between different groups (p<0.001) by one-way ANOVA (Table 2 - see PDF). Comparison of fracture resistance in between the groups by post hoc LSD was also carried out (Table 3 - see PDF).

## Results:

Mean fracture resistance in Group 1(2 mm circumferential ferrule) was 1068.67 ± 107.89, in Group 2(2 mm Buccal and lingual 0.5 mm proximal) was 730.92 ± 82.65, in Group 3(0.5 mm Buccal and lingual 2 mm proximal) was 652.08 ± 71.2, in Group 4(1 mm contra bevel with 4 mm composite into canal) was 853.00 ± 69.76 and in Group 5 (No ferrule) was 402.25 ± 83.16. Mean fracture resistance in Group 1(2 mm circumferential ferrule) was maximum as compared to other groups and minimum in Group 5 (No ferrule). There was highly significant difference in mean fracture resistance in between different groups (p<0.001) by one-way ANOVA.

## Discussion:

The current study compares the different ferrule designs and presence or absence of post in terms of fracture resistance. An ideal post system should have the following features: (a)physical properties similar to dentin, (b)maximum retention with little removal of dentin, (c)distribution of functional stresses evenly along the root surface,(d)esthetic compatibility with the definitive restoration and surrounding tissue, (e)minimal stress during placement and cementation, (f)resistance to displacement, (g)good core retention, (h)easy retrievability (i)material compatibility with core, (j)ease of use, safety and reliability and (k) reasonablecos [18]. Teeth were sectioned 2mm coronal to the CEJ as recommended by Turner [19] to permit embedding of roots with the sectioned surface 2mm above the self-cure resin level corresponding to the position of alveolar bone clinically. This technique permitted standardization of preparation and loading of each sample. Abramovitz *et al.* [20] suggested that 3-6 mm of GP should be left to maintain an apical seal. Post space was prepared as per manufacturer's recommendations. Initially peeso reamer no. 1 was used upto 8mm and later no.2, 3, 4 drills were used in sequence respectively.

## Conclusion:

A uniform 2 mm circumferential ferrule significantly enhances the fracture resistance of endodontically treated teeth. Teeth without a ferrule exhibited the lowest resistance, while non-uniform or reduced ferrule designs offered only partial improvement and were less effective than a complete ferrule. In situations where achieving a full ferrule is not feasible, incorporating a 1 mm contralateral bevel with composite extension into the canal may provide some enhancement in resistance, though it does not match the effectiveness of a full ferrule.
